# Association of Polycyclic Aromatic Hydrocarbon Mixtures with Gestational Iron Deficiency: A Cross-Sectional Analysis of the Zunyi Birth Cohort

**DOI:** 10.3390/nu18213565

**Published:** 2026-10-28

**Authors:** Xiangwen Luo, Min Lu, Derong Fang, Ketao Linghu, Hao Huang, Xinyi Xiao, Shimin Xiong, Rong Zeng, Yijun Liu, Xubo Shen, Yuanzhong Zhou

**Affiliations:** 1 School of Public Health, Zunyi Medical University, Zunyi 563000, China; 18084489055@163.com (X.L.);; 2 Key Laboratory of Maternal & Child Health and Exposure Science, Guizhou Provincial Department of Education, Zunyi 563000, China

**Keywords:** polycyclic aromatic hydrocarbons (PAHs), pregnant women, iron deficiency, PAH metabolites, mixture effect

## Abstract

Background: Maternal iron deficiency is a highly prevalent nutritional disorder during pregnancy and poses substantial risks to both maternal and fetal health. While adequate dietary iron intake and supplementation are critical for maintaining iron balance during pregnancy, emerging evidence suggests that exposure to environmental contaminants, such as polycyclic aromatic hydrocarbons (PAHs), may disrupt iron homeostasis and contribute to iron deficiency. However, epidemiological evidence linking prenatal PAH exposure to gestational iron deficiency remains limited, particularly in populations with low-level exposure. This study aimed to examine the associations of individual and combined PAH exposures during late pregnancy with the odds of iron deficiency. Methods: We conducted a cross-sectional analysis of 1717 pregnant women enrolled in the multicenter Zunyi Birth Cohort and measured ten urinary PAH metabolites. Single-pollutant associations were estimated with weighted quasi-binomial logistic regression using inverse probability weighting (IPW), with restricted cubic spline models used to assess dose–response relationships. Mixture effects were evaluated separately using Bayesian kernel machine regression (BKMR), weighted quantile sum (WQS) regression, and quantile g-computation; these mixture models were fit without IPW. Results: IPW analysis revealed that, compared with pregnant women in the lowest tertile of urinary PAH metabolites, the top three metabolites associated with significantly elevated odds ratios for iron deficiency in those in the highest tertile were 2-OHPhe (OR = 1.96, 95% CI: 1.46–2.63), 3-OHPhe (OR = 1.92, 95% CI: 1.43–2.58), and 1-OHPhe (OR = 1.83, 95% CI: 1.36–2.45). WQS regression found a significant overall mixture index effect (β = 0.28, OR = 1.32, 95% CI: 1.11–1.57, *p* = 0.002), with 3-OHPhe (48.42%), 1-OHPhe (18.49%), and 1-OHPyr (16.53%) accounting for the largest shares of the total mixture weight. Quantile g-computation also detected a significant positive global mixture association (ψ = 0.13, 95% CI: 0.01–0.26, *p* = 0.040), with 3-OHPhe (30.39%), 2-OHPhe (29.56%), and 1-OHPyr (24.02%) as the primary positive contributors; these percentages are based on within-direction normalization and are not directly comparable to the WQS weight percentages. BKMR indicated that the estimated mixture association increased monotonically from slightly negative values at lower quantiles to positive values at higher quantiles. Conclusions: These findings provide novel evidence that exposure to specific PAHs, both individually and as a mixture, particularly phenanthrene derivatives like 3-OHPhe, is associated with increased odds of gestational iron deficiency. Given the cross-sectional design, these findings should be regarded as hypothesis-generating and warrant confirmation in prospective studies.

## 1. Introduction

Iron deficiency is one of the most prevalent nutritional disorders in pregnancy. This condition is primarily attributed to a 2–3-fold increase in maternal iron demand for hemoglobin synthesis and elevated physiological consumption during gestation [1,2]. Characterized by depleted iron reserves and reduced serum ferritin levels, iron deficiency affects approximately 45.6% of pregnant women in China, representing a markedly higher prevalence than that of gestational anemia [3]. Mounting evidence has demonstrated that maternal iron deficiency is closely linked to multiple adverse pregnancy outcomes, including aggravated maternal comorbidities, low birth weight, preterm birth, and intrauterine growth restriction [4,5,6]. For the fetus, decreased maternal serum iron levels restrict transplacental iron transport, which impairs fetal hematopoiesis and oxygen supply. Such disruptions not only increase the risk of intrauterine growth restriction but also may lead to long-term neurodevelopmental deficits in offspring [7,8]. Beyond adverse maternal and fetal consequences, iron deficiency disrupts two core physiological processes: hemoglobin synthesis and cellular energy metabolism. This dual dysfunction drives iron-restricted erythropoiesis and ultimately contributes to the development of gestational anemia [9,10].

Polycyclic aromatic hydrocarbons (PAHs) are ubiquitous persistent organic pollutants widely distributed in air, water, soil, and other environmental matrices, mainly originating from the incomplete combustion of coal, petroleum, and organic materials [11,12,13,14]. Human exposure to PAHs occurs predominantly via inhalation, dermal contact, and dietary ingestion [15]. Accumulating epidemiological and toxicological studies have indicated that PAH exposure is associated with a broad spectrum of health hazards, such as immune dysfunction, respiratory diseases, congenital malformations, childhood cognitive impairment, depressive disorders, cardiovascular diseases, and malignancies [16,17]. Notably, emerging evidence suggests that PAHs can interfere with systemic and cellular iron homeostasis [18,19,20]. PAH metabolites containing oxygenated functional groups are capable of directly chelating intracellular iron and depleting the labile iron pool. The resultant iron complexes further trigger reactive oxygen species (ROS) overproduction through the Fenton reaction [21,22]. Excessive ROS oxidizes serum transferrin, weakens its iron-binding capacity, and consequently disturbs systemic iron circulation [23]. In addition, PAH-induced pulmonary inflammation elevates circulating interleukin-6 (IL-6), initiates the systemic acute-phase response, and upregulates hepatic hepcidin expression [24,25]. Increased hepcidin inhibits ferroportin-mediated iron efflux from macrophages and intestinal epithelial cells, leading to iron sequestration and impaired bioavailability.

To date, the evidence linking PAH exposure to iron status derives mainly from mechanistic and toxicological studies; population-based investigations, particularly among pregnant women, are lacking. Pregnancy is a vulnerable period accompanied by a high prevalence of iron deficiency, which imposes substantial health risks on both mothers and fetuses. Pregnant women are more susceptible to environmental toxicants, including PAHs, due to hormonal fluctuations, elevated metabolic requirements, and increased pulmonary ventilation [26,27,28], which may further exacerbate the odds of iron deficiency. Therefore, exploring the association between PAH exposure and iron metabolism among pregnant women is of important public health significance. Previous mechanistic studies have suggested that PAHs can disrupt iron homeostasis by modulating hepcidin expression [29,30,31]. However, direct human epidemiological evidence linking PAH exposure to iron deficiency remains limited. In particular, few studies have clarified the effects of PAHs on systemic iron status among pregnant residents in low-PAH-exposure regions, and the health impacts of chronic low-dose PAH exposure on maternal iron reserves are still poorly understood.

To fill this research gap, the present study was conducted based on the Zunyi Birth Cohort [32], an area with relatively low ambient PAH exposure. Focusing on pregnant women as a susceptible population, we aimed to explore whether low-level prenatal PAH exposure is associated with altered iron metabolism and iron deficiency. Ten urinary PAH metabolites were determined, and iron status was assessed by serum ferritin measurement. Restricted cubic spline models were applied to evaluate dose–response relationships, while Bayesian kernel machine regression, weighted quantile sum regression, and quantile g-computation models were used to assess mixture effects. Inverse probability weighting was further adopted to control potential confounding factors. The findings of this study will enrich the epidemiological evidence on PAH-related health risks and provide scientific references for environmental pollution prevention and targeted nutritional intervention strategies for pregnant women.

## 2. Materials and Methods

### 2.1. Study Population

This study was conducted based on the Zunyi Birth Cohort, which was established in Southwest China from May 2020 to April 2022 and initially enrolled 6583 pregnant women. Participants were recruited from four medical institutions, including the Affiliated Hospital of Zunyi Medical University, the Second Affiliated Hospital of Zunyi Medical University, Meitan County People’s Hospital, and Xishui County People’s Hospital. Participants from the cohort were further screened according to the following criteria: ① spontaneous conception; ② singleton pregnancy; ③ permanent local residency with no relocation plan in the near future; ④ availability of urine biological samples; ⑤ completion of on-site face-to-face questionnaire interviews.

**Figure 1:**
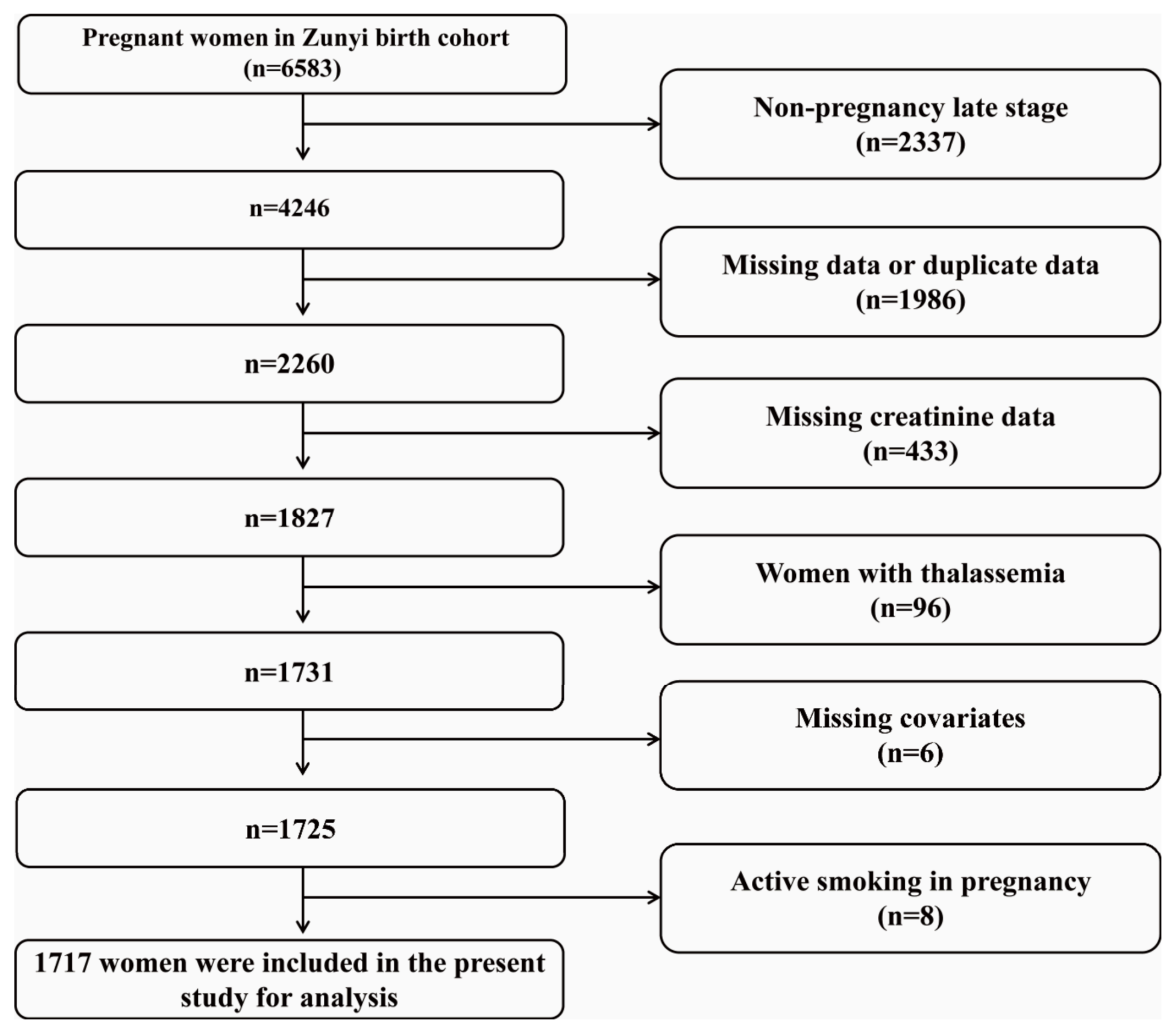
Study profile, showing the creation of the study sample from the Zunyi Birth Cohort.

At the initial screening stage, 2337 participants who provided urine samples for pollutant testing during the first or second trimester (i.e., gestational age <28 weeks and thus not in late pregnancy) were excluded. Subsequently, 1986 participants with missing data for PAHs, 433 individuals with missing serum creatinine data, 96 individuals diagnosed with thalassemia, 6 individuals with incomplete information on key covariates, and 8 pregnant women with a history of active smoking during pregnancy were excluded, yielding a final analytic sample of 1717 pregnant women (Figure 1). All participants provided written informed consent prior to enrollment. The study protocol was approved by the Ethics Committee of Zunyi Medical University (Approval No. [2019] H-005).

### 2.2. Iron Deficiency Assessment

After 28 weeks of gestation, the participants had their venous blood samples collected after fasting at night. Serum ferritin concentrations were quantified by hospital clinical laboratories. Gestational iron deficiency in this study was defined as serum ferritin <30 μg/L, consistent with widely accepted cutoffs for depleted maternal iron stores. No hemoglobin threshold was applied to exclude participants [33,34,35,36].

### 2.3. Urinary PAHs Metabolites Measurement

Urine samples were collected via a random spot sampling method by trained personnel and stored at −80 °C until subsequent quantitative analysis. Urinary metabolites of polycyclic aromatic hydrocarbons (PAHs) were quantified using gas chromatography–tandem mass spectrometry (GC–MS/MS) (Agilent 7010B, USA). The target metabolites included 1-hydroxynaphthalene (1-OHNap), 2-hydroxynaphthalene (2-OHNap), 2-hydroxyfluorene (2-OHFlu), 9-hydroxyfluorene (9-OHFlu), 1-hydroxyphenanthrene (1-OHPhe), 2-hydroxyphenanthrene (2-OHPhe), 3-hydroxyphenanthrene (3-OHPhe), 4-hydroxyphenanthrene (4-OHPhe), 9-hydroxyphenanthrene (9-OHPhe), and 1-hydroxypyrene (1-OHPyr). Detailed analytical procedures have been described previously [37]. Briefly, each urine sample (1.5 mL) was mixed with sodium acetate buffer (1 mL), internal standard (10 μL), and β-glucuronidase/sulfatase (10 μL), followed by overnight incubation (12–16 h) at 37 °C. Subsequently, saturated MgSO_4_·7H_2_O (2.1 g) was added. The mixture was subjected to triple liquid–liquid extraction with hexane (4:1, v/v), and the supernatant was collected into a new 5 mL centrifuge tube after each extraction. The combined supernatant was concentrated to approximately 150 μL under a gentle nitrogen stream, transferred to an inner tube, and dried completely under nitrogen. For derivatization, a silylation reagent (100 μL) was added, and the mixture was heated at 90 °C for 45 min. After cooling to room temperature, the derivatized sample was analyzed by GC–MS/MS.

To account for variations in urine dilution, PAH metabolite concentrations were normalized to urinary creatinine levels using the formula below: creatinine-adjusted PAH concentration (μg/g creatinine) = PAH concentration (μg/L)/[urinary creatinine concentration (mmol/L) × 113.12 (g/mol) × 10^−3^]. Creatinine normalization was adopted as the primary approach, and three additional sensitivity analyses were performed to evaluate the robustness of the associations (Supplementary Materials, Figure S1): (1) Unadjusted (raw) PAH concentrations; (2) Raw PAH concentrations with urinary creatinine included as a covariate in the regression model; and (3) Specific-gravity-corrected PAH concentrations (C_corr = C × (SG_ref − 1)/(SG − 1), with SG_ref = 1.020).

### 2.4. Quality Control

For quantitative analysis, chemical metabolites were assessed using the standard addition method. Three different concentrations (low, medium, and high) of the standard solution were separately spiked into 1.5 mL of urine, with each concentration tested in eight replicates to determine recovery and precision. The limit of detection (LOD) was defined as a signal-to-noise ratio (S/N) of 3. At least one procedural blank was included in each batch of twenty urine samples, and the relative deviation between duplicate samples was required to be ≤20%. In each analytical run, the internal standard was monitored, and its response was maintained within ±30% of the corresponding calibration curve response.

### 2.5. Covariates

Trained investigators collected data from pregnant women via face-to-face interviews. The following variables were included: age (years), educational attainment (junior high school or below, senior high school, college or above), employment status (employed/unemployed), annual household income (<¥100,000, ¥100,000–¥150,000, ≥¥150,000, or unknown), pre-pregnancy exercise (yes/no), alcohol consumption during pregnancy (yes/no), passive smoking exposure during pregnancy (yes/no), use of nutritional supplements during pregnancy (e.g., folic acid, iron, vitamins, vitamin B12; yes/no), gestational weeks, and estimated weekly dietary intake of iron and calcium (g/week) based on a food frequency questionnaire. As part of the same food frequency questionnaire, participants also reported how frequently they consumed grilled or smoked foods; this variable was reserved for evaluation as an additional covariate in a planned sensitivity analysis. The dietary nutrient intake was calculated using the following formula:

(1) $$\mathrm{Nutrient}\;\mathrm{intake}\;(g/\mathrm{week})\;=\;\Sigma_{i}F_{i}\times P_{i}\times C_{i}$$

In this formulation, F_i_ denotes the weekly consumption frequency of the *i*-th food item, as obtained from questionnaire responses (e.g., “3 times/week”). P_i_ represents the standard portion weight, which is defined based on the Food Frequency Questionnaire or the China Food Composition Table. C_i_ is the corresponding nutrient content, derived from standard food composition tables. Pre-pregnancy body mass index (BMI) was calculated as weight (kg) divided by height squared (m^2^).

### 2.6. Statistical Analysis

Concentrations below the LOD were primarily imputed as LOD/√2. Two sensitivity analyses were additionally performed to assess the robustness of the findings: one using random imputation from a uniform distribution between zero and the LOD, and another using weighted logistic regression in which non-detectable observations were down-weighted to 0.6 to reflect the greater uncertainty associated with left-censored data.

As an additional sensitivity analysis, all ten metabolites were modeled as binary detected/non-detected variables, rather than the tertile-based classification used in the primary analysis, to assess whether the observed associations were robust to alternative exposure coding (Supplementary Materials, Table S1b). Continuous analyses used log-transformed creatinine-normalized concentrations. Inter-metabolite correlations were assessed using Spearman’s rank correlation. Two additional sensitivity analyses were performed: one adding hospital site as a covariate, and another adding grilled/smoked food intake frequency from the FFQ. In both analyses, the results were consistent with the primary findings (Supplementary Materials, Table S1c).

To reduce measured confounding, we used a two-step stabilized inverse probability weighting (IPW) approach, run independently for each of the 10 PAH metabolites.

In the first step, creatinine-normalized metabolite concentration was split into tertiles (T1 = lowest, T2 = middle, T3 = highest, T1 as reference). A joint multinomial logistic regression was fit with tertile group as the dependent variable, adjusted for maternal age, pre-pregnancy BMI, education, occupation, household income, passive smoking, exercise, drinking status, nutritional supplementation, dietary iron intake, and gestational week. For each participant, we calculated the conditional probability of belonging to their observed tertile (propensity score), then derived stabilized IPW weights as the ratio of the marginal to the covariate-adjusted probability of their observed tertile. Weights were capped at the 1st and 99th percentiles to reduce the influence of extreme values, and covariate balance across tertiles was assessed with standardized mean differences (SMDs) before and after weighting.

In the second step, metabolite-specific capped weights were incorporated as survey weights in a weighted quasi-binomial logistic regression model, with binary iron deficiency status as the outcome and tertile group as the main predictor. Survey (linearized) standard errors, rather than model-based standard errors, were used to account for the weighting. This model estimated odds ratios (ORs) and 95% confidence intervals for T2 vs. T1 and T3 vs. T1; linear trend *p*-values were calculated by modeling tertile group as a continuous variable. Per-metabolite weight distributions, SMDs, and effective sample sizes are provided in Tables S4 and S5.

$$\ln\frac{P(T=k|C)}{P(T=1|C)}=\alpha_{k}+\sum_{j}\gamma_{kj}C_{j},\;k=T2,T3\;$$

$$SW_{i}=\frac{P(T=t_{i})}{P(T=t_{i}|C_{i})}$$

where **C** is the covariate vector; for k ∈ {T2, T3}, α_k and γ_kj are the multinomial intercepts and coefficients; the numerator P(T = t_i) is the marginal probability of the observed tertile and the denominator is its covariate-conditional (fitted) probability.

We conducted restricted cubic splines (RCS) to assess the univariate and nonlinear associations between PAH metabolites and iron deficiency. Dose–response relationships were further characterized with RCS based on continuous log-transformed concentrations. All trend *p*-values were corrected for multiple testing by controlling the false discovery rate. The overall and nonlinearity *p*-values from the RCS analyses were reported as raw values, as these were considered exploratory analyses.

To evaluate the joint effect of PAH co-exposure, we applied Bayesian kernel machine regression (BKMR). Because strong pairwise correlations among urinary OH-PAH metabolites (Supplementary Materials, Figure S2) could destabilize Markov chain Monte Carlo variable selection, we implemented group-wise variable selection in a single BKMR model. Metabolites were assigned to two groups in a data-driven manner based on the observed Spearman correlation structure (Figure S2), placing metabolites with stronger pairwise correlations in the same group to reduce multicollinearity and stabilize variable selection. Group 1 comprised 1-OHNap, 9-OHPhe, and 1-OHPyr; Group 2 comprised 2-OHNap, 2-OHFlu, 9-OHFlu, 1-OHPhe, 2-OHPhe, 3-OHPhe, and 4-OHPhe. Metabolites within each group shared a common inclusion indicator, and all ten were modelled simultaneously. The model used a probit link with z-scored exposures and was fit with 10,000 MCMC iterations. For the binomial (probit) model, BKMR internally discards the first 50% as burn-in and stores only the remaining 5000 draws; these stored draws were used for posterior inference, with no additional thinning. Default BKMR priors were used: a Gaussian kernel with Normal coefficient priors, Bernoulli (0.5) group-inclusion priors, and an inverse-gamma residual variance prior. Convergence was assessed by visual inspection of trace plots, the effective sample size of the posterior draws, and comparison of posterior summaries between the first and second halves of the retained chain. Posterior inclusion probabilities were estimated per group and metabolite; weighted quantile sum (WQS) regression (gWQS package) used a positive direction constraint (b1_pos = TRUE), non-negative weights (b_constr = TRUE), a 40%/60% training/validation split (validation = 0.6), 10,000 bootstrap iterations (b = 10,000), and quartile binning (q = 4). Quantile g-computation (qgcomp package) used quartile binning, 10,000 bootstrap iterations, and Bayesian estimation (bayes = TRUE); positive and negative component weights were normalized separately within each direction, each weight being |coefficient|/Σ|coefficients within that direction| × 100, so that positive weights summed to 100% and negative weights summed to 100%. All mixture models were adjusted for maternal age, pre-pregnancy BMI, education, occupation, income, passive smoking, exercise, drinking, nutrition, iron intake, and gestational week. The mixture models were fitted without inverse probability weighting; IPW was applied only in the single-pollutant tertile-based analysis. Analyses were performed in R 4.4.2 with two-sided *p* < 0.05.

## 3. Results

### 3.1. Characteristics of the Study Population

A total of 1717 pregnant women were included in the analysis (Figure 1). The mean age was 27.01 years, the median gestational age at blood and urine sampling was 38 weeks (IQR: 33–39 weeks), and the prevalence of iron deficiency was 22.3% (Table 1). The basic characteristics of the 2529 women who did not meet the inclusion criteria are presented in Table 2, among whom 344 (13.6%) had iron deficiency. Inter-metabolite correlations were assessed using Spearman’s rank correlation (Supplementary Materials, Figure S2). Among the ten measured PAH metabolites, the detection rates ranged from 49.2% (4-OHPhe) to 76.6% (2-OHFlu), which also exhibited the highest median creatinine-adjusted concentration (Supplementary Materials, Table S3a). The limits of detection, spike recovery, and intra-/inter-day precision for each metabolite are summarized in Table S3b.

### 3.2. Individual PAH Metabolites and Iron Deficiency Odds

**Figure 2:**
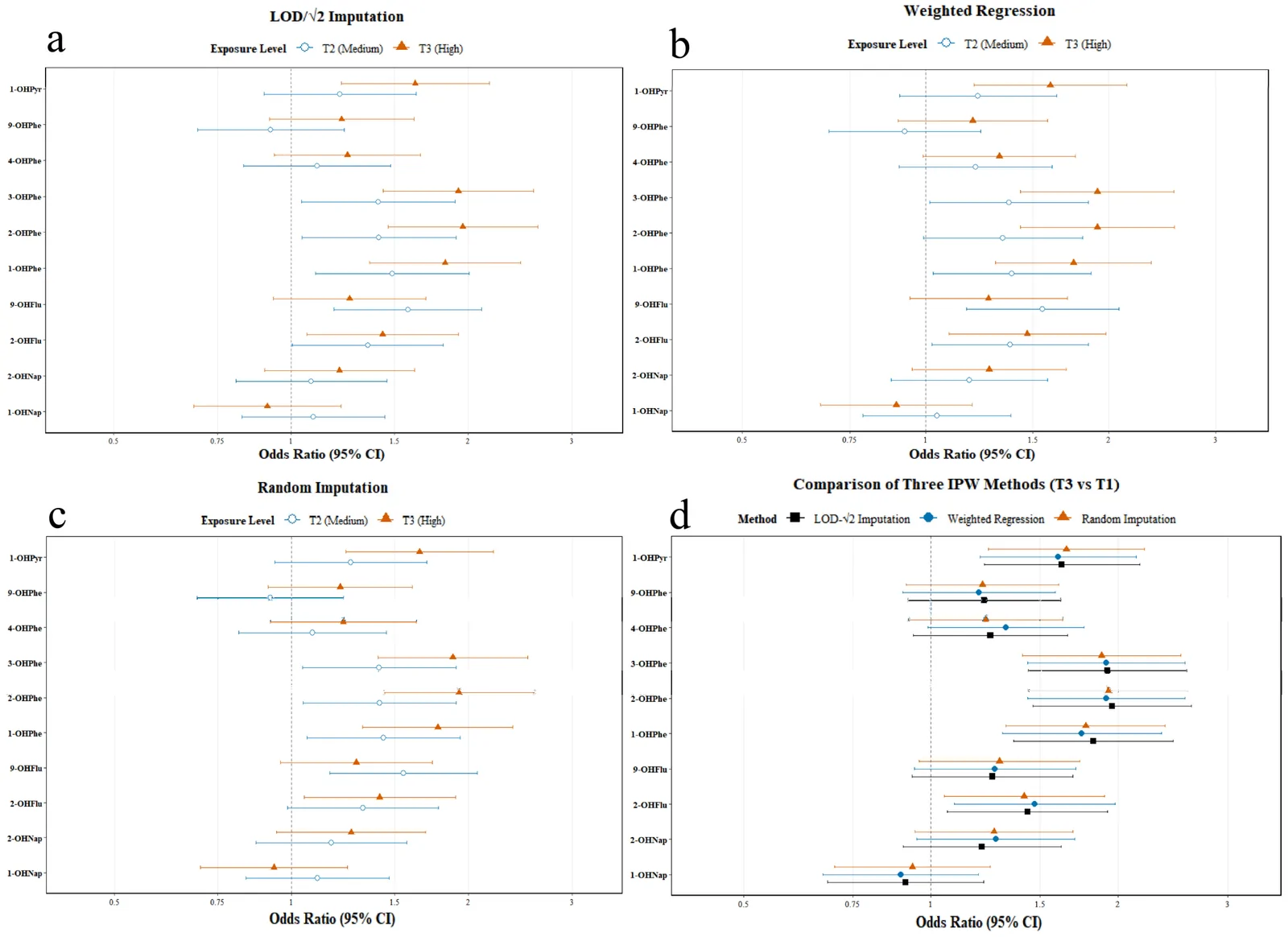
Association between urinary PAH metabolites and gestational iron deficiency under three approaches for handling values below the limit of detection (LOD), all analyzed with inverse probability weighting (IPW): (**a**) primary analysis using LOD/√2 substitution; (**b**) sensitivity analysis using weighted logistic regression with non-detectable observations down-weighted to 0.6; (**c**) sensitivity analysis using random imputation below the LOD; (**d**) comparison of odds ratios across the three approaches.

**Figure 3:**
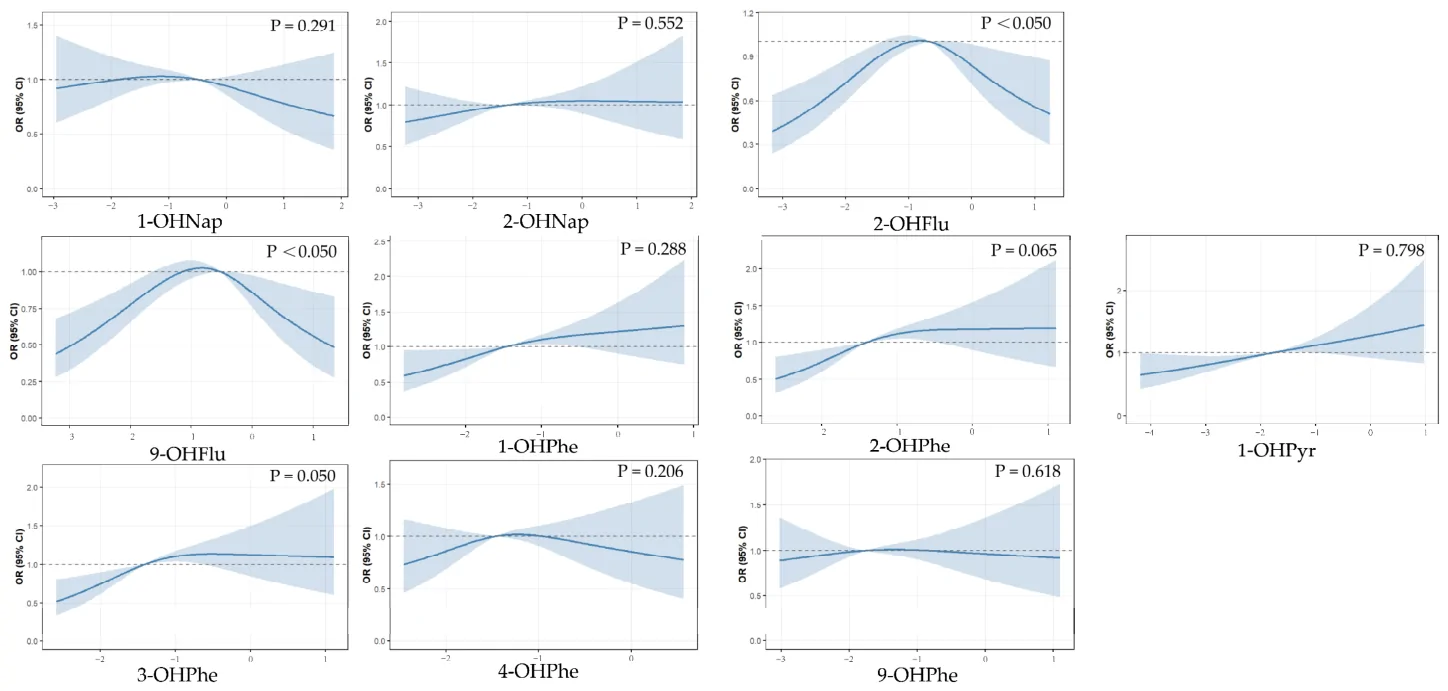
A restricted cubic spline plot of polycyclic aromatic hydrocarbon metabolites and their association with iron deficiency during pregnancy.

Across the 10 independent metabolite-specific fits, stabilized weights were mild, ranging from 0.43 to 2.23 (median: 0.97; IQR: 0.81–1.14), with a median effective sample size of approximately 1636 (Table S4). Weighting reduced the maximum SMD across exposure tertiles from 0.42 to 0.22 (Table S5); although the weighted maximum SMD slightly exceeded the conventional 0.10 threshold, the mild weights and the consistent sensitivity analyses indicate that any residual imbalance had little influence on the estimates. In the primary IPW logistic regression (LOD/√2 imputation), five metabolites in the highest tertile (T3) were significantly associated with higher odds of iron deficiency versus the lowest tertile (T1): 1-OHPhe 1.83 (95% CI: 1.36–2.45), 2-OHPhe 1.96 (95% CI: 1.46–2.63), 3-OHPhe 1.92 (95% CI: 1.43–2.58), 1-OHPyr 1.63 (95% CI: 1.22–2.17), and 2-OHFlu 1.43 (95% CI: 1.06–1.92) (Figure 2). All five associations remained significant after Benjamini–Hochberg correction (FDR q < 0.05) and were robust across three LOD-handling strategies (LOD/√2 imputation, weighted regression, and random imputation) (Supplementary Materials, Table S1a). Restricted cubic spline analysis (Figure 3; Supplementary Materials, Table S2) further showed heterogeneous dose–response patterns: 1-OHPhe, 2-OHPhe, 3-OHPhe, and 1-OHPyr showed significant linear positive associations (3-OHPhe borderline nonlinear, *p* for nonlinearity = 0.050), 2-OHFlu and 9-OHFlu showed significant inverted U-shaped associations, and the remaining four metabolites showed no significant association. The *p*-values shown in Figure 3 are the *p*-values for nonlinearity; the overall *p*-values are provided in Table S2.

### 3.3. Key PAH Components and Their Contribution Profiles Associated with Gestational Iron Deficiency

**Figure 4:**
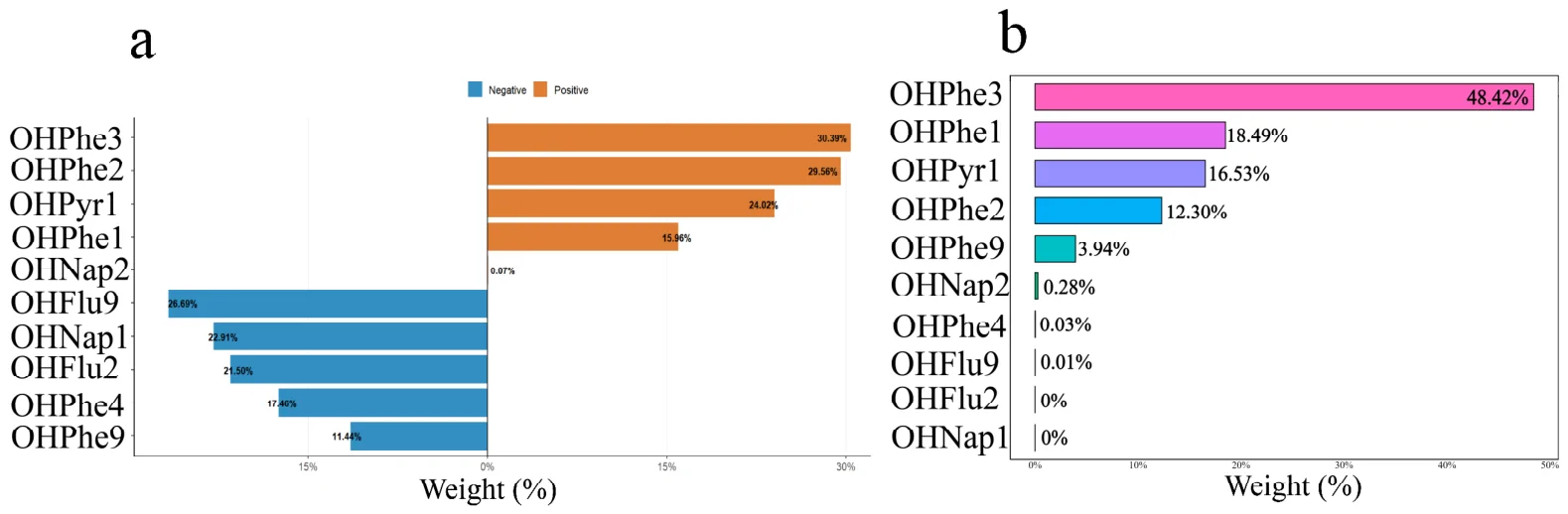
Weight contributions of the ten PAH metabolites. (**a**) Quantile g-computation weights for the positive (harmful) and negative (inverse) directions, reported as percentages within each direction (the positive and negative weights each sum to 100%); 3-OHPhe, 2-OHPhe and 1-OHPyr were the largest positive contributors, and 9-OHFlu, 1-OHNap and 2-OHFlu the largest negative contributors. (**b**) Weighted quantile sum (gWQS) weights, which sum to 100% across the whole mixture, with 3-OHPhe as the largest contributor (48.42%). The two panels use different normalization bases—within-direction for (**a**) and whole-mixture for (**b**)—so their percentages are not directly comparable.

Q-g analysis revealed a statistically significant positive association of the PAH mixture with iron deficiency (ψ = 0.13, 95% CI: 0.01–0.26, *p* = 0.040). Within the mixture, 3-OHPhe (30.39%), 2-OHPhe (29.56%), 1-OHPyr (24.02%), 1-OHPhe (15.96%), and 2-OHNap (0.07%) emerged as the positive (i.e., harmful) contributors, while 9-OHFlu (26.69%), 1-OHNap (22.91%), 2-OHFlu (21.50%), 4-OHPhe (17.46%), 9-OHPhe (11.44%) showed negative weights (Figure 4a). In quantile g-computation, the positive and negative weights were normalized separately, so each percentage is a relative contribution within its own direction (the positive weights sum to 100%, and the negative weights sum to 100%), rather than within the whole mixture. These values are therefore not directly comparable to the WQS weights, which are normalized across the entire mixture (e.g., 48.42% for 3-OHPhe). WQS regression revealed a statistically significant positive association between the PAH mixture index and gestational iron deficiency (β = 0.28, OR = 1.32, 95% CI: 1.11–1.57, *p* = 0.002). WQS regression further highlighted the dominant role of 3-OHPhe, which accounted for 48.42% of the total mixture weight, followed by 1-OHPhe (18.49%) and 1-OHPyr (16.53%) (Figure 4b).

### 3.4. PAH Mixture Associations

**Figure 5:**
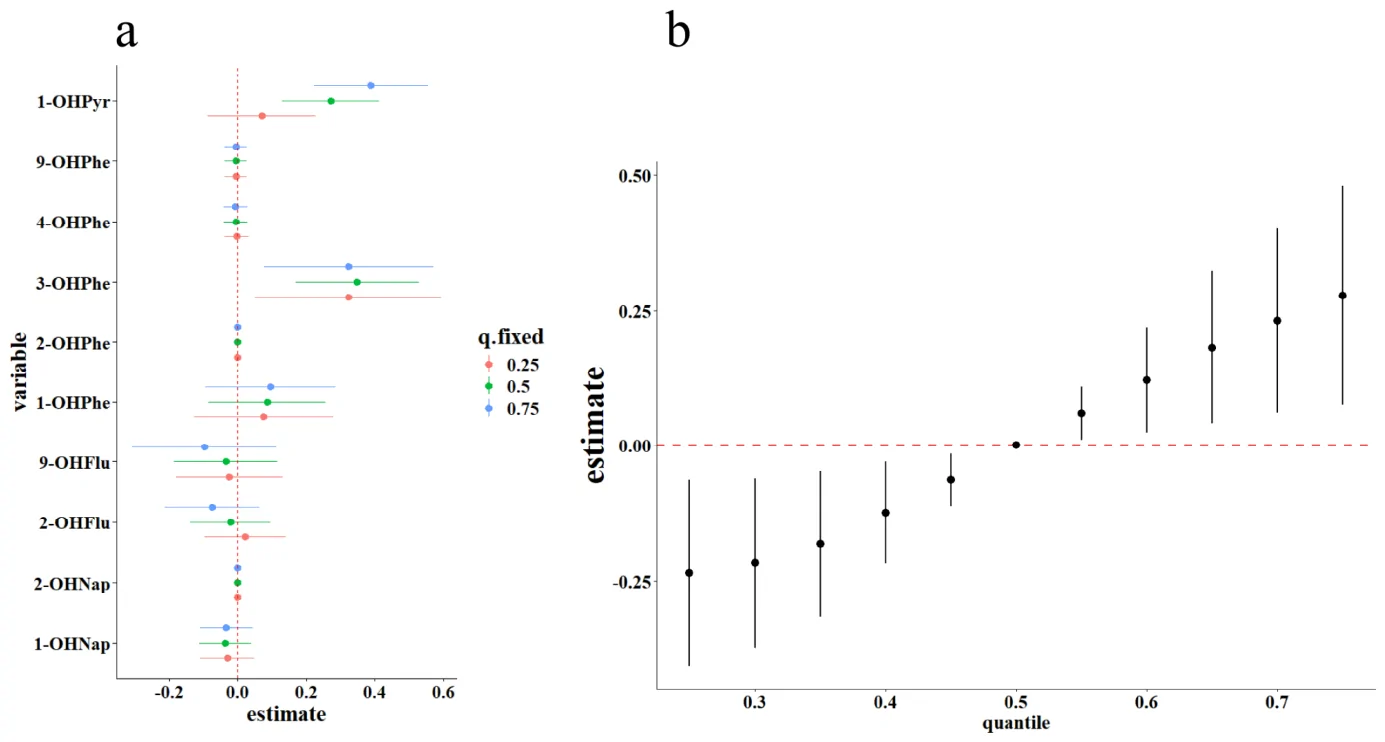
(**a**) Conditional associations of individual metabolites, with co-exposures fixed at the 25th, 50th, and 75th percentiles; (**b**) Quantile-specific associations of the overall PAH mixture.

The group-wise posterior inclusion probabilities were high for both groups (0.979 for Group 1 and 0.998 for Group 2), supporting the joint inclusion of all ten metabolites in the model; within groups, 1-OHPyr (conditional PIP = 0.989) and 3-OHPhe (conditional PIP = 0.828) had the highest conditional inclusion probabilities. The effective sample sizes of the retained draws were 2674–4215 for the regression coefficients (mean 3578) but were lower for the kernel smoothness parameters (r; 39–645, mean 214) and the variable-selection indicators (delta; 36–825, mean 224); posterior summaries were consistent between the first and second halves of the retained chain. The estimated association of 1-OHPyr (75th vs. 25th percentile) strengthened as the other co-exposures were fixed at increasing quantiles and was largest when they were fixed at the 75th percentile (Figure 5a). The single-exposure dose–response functions for all 10 individual PAH metabolites are presented in Supplementary Materials, Figure S3. The pairwise joint marginal effects of the metabolites are presented in Supplementary Materials, Figure S4. The estimated overall mixture exposure-response relationship, in which all metabolites were set to the same quantile, showed a monotonic directional trend across quantiles (Figure 5b). Across the lower quantile range (0.25 to 0.45), estimated associations with iron deficiency were consistently negative, with all 95% confidence intervals lying entirely below the null. These negative estimates simply reflect comparison of the mixture at lower quantiles against the median reference and should not be interpreted as protective effects. The association was anchored at zero at the 0.5 reference point, and first reached statistical significance in the positive direction at the 0.55 quantile (estimate = 0.056, 95% CI: 0.008–0.105). The magnitude of this positive association increased steadily and monotonically across higher quantiles, reaching an estimate of 0.267 (95% CI: 0.065–0.470) at the 0.75 quantile. Furthermore, the bivariate exposure–response analysis suggested a non-additive (synergistic) pattern between 1-OHPyr and 3-OHPhe, with the estimated association of each metabolite increasing with the quantile of the co-metabolite (Supplementary Materials, Figure S5). This interaction was inferred from the separation of the dose–response curves across quantiles of the co-metabolite, consistent with a statistical interaction suggested by the nonparametric model rather than an association quantified on an additive scale.

## 4. Discussion

To our knowledge, direct epidemiological evidence linking urinary PAH metabolites to gestational iron deficiency remains sparse: previous human studies have largely characterized PAH exposure levels in pregnant populations or examined associations with oxidative stress biomarkers, rather than iron status outcomes. The present cross-sectional analysis addresses this gap by examining associations between prenatal PAH exposure and elevated odds of iron deficiency in late pregnancy. Five metabolites (1-OHPhe, 2-OHPhe, 3-OHPhe, 2-OHFlu, and 1-OHPyr) were significantly associated with higher odds of iron deficiency; among these, 1-OHPhe, 3-OHPhe, and 1-OHPyr were the dominant statistical contributors to the positive mixture index in the WQS model. Furthermore, the overall mixture associations may be modulated by component interactions; a potential non-additive interaction pattern between 3-OHPhe and 1-OHPyr was suggested.

In the WQS regression analysis, 3-OHPhe exhibited the highest contributing weight to the odds of iron deficiency, followed by 1-OHPhe and 1-OHPyr. The biological plausibility of these metabolites as statistical contributors to iron deficiency is supported by existing toxicological mechanisms. As the parent compound of 3-OHPhe and 1-OHPhe, phenanthrene can robustly induce oxidative stress [38], exacerbate the basal oxidative burden during pregnancy, and may upregulate IL-6 expression [39]. Epidemiological studies have demonstrated that PAH exposure is linked to elevated circulating pro-inflammatory cytokines, including IL-6, in general populations [24], and pregnant women are expected to be similarly susceptible given their heightened inflammatory response to environmental toxicants [26,27,28]. IL-6 is a potent stimulator of hepatic hepcidin synthesis via the JAK2/STAT3 signaling pathway [25,40]. In normal pregnancy, serum hepcidin concentrations decline progressively across gestation to accommodate the markedly increased iron demand of the expanding fetus–placenta unit [41]. This physiological decline is a critical adaptation that enhances intestinal iron absorption and mobilizes iron from maternal stores. However, we speculate that PAH-associated chronic inflammation may counteract this physiological decline in hepcidin. If IL-6 levels remain inappropriately elevated due to sustained PAH exposure, hepatic hepcidin synthesis may be persistently upregulated, resulting in hepcidin concentrations that are higher than expected for the gestational stage. Elevated hepcidin binds to and induces the internalization and degradation of ferroportin, the sole known cellular iron exporter expressed on duodenal enterocytes, macrophages, and hepatocytes [42,43]. The resulting blockade has two major consequences: (a) impaired dietary iron absorption at the duodenal mucosa, and (b) impaired iron recycling from senescent red blood cells by macrophages [44,45]. Together, these effects impose a state of chronic iron restriction, in which iron influx into the circulation is insufficient to meet the escalating demands of pregnancy. Over the course of gestation, this sustained iron restriction—driven by inappropriately elevated hepcidin—may progressively erode maternal iron stores. Even though acute inflammation typically elevates both hepcidin and ferritin as part of the acute-phase response [46], the unique context of pregnancy may alter this trajectory. If hepcidin-mediated blockade of iron absorption and recycling persists throughout gestation, the cumulative chronic iron deficit may eventually overwhelm the acute-phase ferritin response, leading to net iron store depletion manifesting as low serum ferritin. Consequently, phenanthrene may promote hepcidin synthesis, which could suppress intestinal iron absorption and macrophage-mediated iron recycling, ultimately triggering functional iron deficiency [40,47,48,49]. Meanwhile, it directly disrupts iron metabolism and erythropoiesis, further synergizing the progression of iron deficiency [50,51,52,53].

This interpretation is supported by evidence from obese pregnant women, who exhibit persistent low-grade chronic inflammation accompanied by decreased serum ferritin and paradoxically elevated hepcidin—a pattern consistent with hepcidin-mediated impairment of iron absorption [54]. Furthermore, Weiss and Goodnough described a recognized continuum between pure iron deficiency and anemia of chronic disease, in which prolonged inflammation-driven iron restriction can ultimately deplete body iron stores, producing a “mixed” pattern with characteristics of both conditions [46]. However, it is important to emphasize that none of these intermediate biomarkers were measured in the present study. Therefore, the oxidative stress → IL-6 → hepcidin → ferroportin pathway should be interpreted as a plausible mechanistic hypothesis generated by the integration of our epidemiological findings with the previous experimental literature, rather than as a mechanism demonstrated in this cohort. Future studies with concurrent measurement of these intermediate biomarkers are needed to validate this proposed pathway.

Q-g analysis in the present study revealed that certain PAH metabolites (e.g., 2-OHFlu and 9-OHFlu) exhibited potential “protective effects”. However, this result should be interpreted with caution. The observed “protective effect” may be attributable to statistical artifacts or residual confounding. First, the overall average weighting adopted in the Q-g model may mask authentic dose-dependent variations, leading to the misinterpretation of non-significant toxic effects at low exposure levels as protective associations, while the toxic effects remain prominent at high PAH exposure levels. Negative weights in quantile g-computation represent conditional inverse statistical associations after adjustment for co-exposures, and should not be interpreted as biological protective effects. They may arise from collinearity among metabolites or residual confounding, and are not attributable to the weight normalization, which was applied separately within each direction. Second, unmeasured or insufficiently adjusted confounders, including dietary antioxidant intake, co-existing environmental pollutants, genetic susceptibility, and collinearity among PAH metabolites, may distort the true association between PAH exposure and iron status. It is also important to note that the positive association observed for 2-OHFlu in the single-pollutant IPW models (T3 vs. T1, OR = 1.43, 95% CI: 1.06–1.92) and its negative weight in quantile g-computation are not contradictory: the qgcomp weight is a conditional contribution estimated after mutual adjustment for the other nine metabolites, whereas the single-pollutant OR is a marginal estimate. In the presence of strong inter-metabolite correlations, marginal and conditional estimates can differ in direction; the negative weight should therefore be interpreted in line with the conditional inverse statistical association discussed above, while the marginal positive OR may partly reflect confounding by correlated co-exposures (e.g., 3-OHPhe and 1-OHPhe).

In the BKMR analysis, when all PAH metabolites were set to the same quantile, the estimated association with iron deficiency increased monotonically. The positive association at the highest exposure levels should be interpreted with caution given the wider CIs and fewer observations. The bivariate BKMR analysis suggested a qualitative non-additive pattern between 1-OHPyr and 3-OHPhe (Supplementary Materials, Figure S5); because BKMR is nonparametric, this should be interpreted as a statistical pattern rather than evidence of biological synergy. The hypothesis that co-exposure to these metabolites may amplify oxidative stress and inflammatory responses is derived from previous experimental literature [55,56] rather than from direct measurements in the present cohort, in which no oxidative-stress, inflammatory, or iron-regulatory biomarkers were assessed.

This cross-sectional analysis embedded within a multicenter birth cohort provides novel epidemiological evidence linking exposure to polycyclic aromatic hydrocarbons (PAHs) with increased odds of iron deficiency in late pregnancy (after 28 weeks of gestation), addressing a significant knowledge gap. A key strength lies in the application of cutting-edge mixture modeling methods—including BKMR, WQS regression, and quantile g-computation. This robust analytical framework allowed us to disentangle the complex combined effects of real-world PAH co-exposure, identify 3-OHPhe as the dominant statistical contributor to the positive WQS mixture index, and suggest a potential non-additive interaction pattern between 1-OHPyr and 3-OHPhe. The study further characterized heterogeneous dose–response relationships, ranging from linear to inverted U-shaped patterns. The large sample size (n = 1717) from a well-defined birth cohort and the use of several sensitivity analyses employing alternative approaches to handle non-detectable values (LOD/√2 substitution, random imputation, and weighted regression) strengthen the internal validity of our findings within this population.

From a public health perspective, the dominant statistical contribution of 3-OHPhe in the WQS model, a phenanthrene metabolite associated with combustion-related PAH exposure [57,58], suggests that reducing residential combustion-derived PAH exposure may be relevant for maternal iron status, although specific emission sources were not assessed in this study. These strategies should therefore be viewed as hypothesis-generating and require prospective evaluation.

However, several limitations of this study should be acknowledged. First, a single third-trimester spot urine sample likely captures recent rather than cumulative pregnancy PAH exposure; although urinary PAH metabolites show reasonable short-to-medium reproducibility, their representation of earlier gestation remains uncertain. Low detection rates for some metabolites (e.g., 4-OHPhe: 49.2%; 2-OHFlu: 76.6%) may introduce measurement error; despite LOD/√2 imputation and alternative-censoring sensitivity analyses, such findings warrant caution. Second, iron deficiency was defined solely by serum ferritin <30 μg/L. Although ferritin is the most sensitive iron-store indicator, it is an acute-phase reactant; CRP and other inflammatory markers were not measured, precluding inflammation-adjusted sensitivity analyses—particularly relevant given the hypothesized IL-6/hepcidin pathway. Additionally, serum ferritin was measured independently in the routine clinical laboratories of the four participating hospitals, without formal cross-site calibration or harmonized quality control, which may introduce site-related outcome measurement error. However, adjustment for hospital as a categorical covariate did not materially alter the primary associations, suggesting that site differences did not drive the findings. Third, residual confounding cannot be excluded: the dataset lacked folic acid/multivitamin use and detailed iron-supplement data (type, dose, duration, and adherence), and parity was excluded due to excessive missing values. Although dietary iron intake and supplementation were adjusted for, key determinants of iron bioavailability (heme/non-heme proportions, dietary enhancers, and inhibitors) were not captured. The stabilized propensity-score IPW improved covariate balance across exposure tertiles (maximum SMD reduced from 0.42 to 0.22), addressing confounding rather than selection; residual imbalance (mainly for household income) may still leave some residual confounding. Separately, iron-deficiency prevalence was lower among excluded (13.6%, 344/2529) than included (22.3%, 383/1717) women; because the IPW does not model selection into the analytic sample, it does not correct this, and residual selection bias cannot be fully excluded. E-values for unmeasured confounding were calculated (Table S6). For the single-pollutant estimates, E-values ranged from 1.68 to 2.15 (1.20–1.71 for the CI bounds); for the mixture estimates, they were 1.34 (1.06) for quantile g-computation and 1.56 (1.29) for WQS. These values indicate the minimum joint association an unmeasured confounder would need with both exposure and outcome to move an estimate to the null; the borderline mixture estimates are correspondingly more sensitive, supporting a hypothesis-generating interpretation. Fourth, the large number of tests raises false-positive concerns; FDR correction was applied only to primary individual-metabolite trend tests. Additionally, in the BKMR model, the effective sample size was low for some variable-selection indicators (as low as 36): these binary indicators are strongly autocorrelated and remain at zero for metabolites that are rarely selected. Because the posterior inclusion probabilities are posterior means of these indicators, PIPs for weakly selected metabolites carry greater Monte Carlo uncertainty; we therefore do not claim formal adequate convergence, and the BKMR findings are treated as qualitative and hypothesis-generating. Fifth, as a cross-sectional analysis within a birth cohort, PAH metabolites and iron status were measured simultaneously at a single time point, which precludes definitive causal inference; the generalizability of our findings to populations with different exposure profiles or earlier gestation periods also requires further investigation. Our findings should therefore be considered hypothesis-generating and require independent cohort confirmation.

## 5. Conclusions

These findings suggest that exposures to five PAH metabolites—1-OHPhe, 2-OHPhe, 3-OHPhe, 2-OHFlu, and 1-OHPyr—in late pregnancy are significantly associated with higher odds of gestational iron deficiency. The three mixture approaches converged on positive associations at higher exposure levels—WQS and qgcomp detected statistically significant positive global mixture associations, and BKMR indicated that the estimated association became positive at higher quantiles—with 3-OHPhe emerging as the dominant statistical contributor in WQS and qgcomp. Collectively, these PAH metabolites—particularly phenanthrene derivatives such as 3-OHPhe—may be relevant markers for maternal iron status. These findings suggest that residential combustion-derived PAH exposure may be a relevant correlate of maternal iron status, though this cross-sectional study does not establish a causal relationship and requires prospective confirmation.

## Figures and Tables

**Table 1:** Basic characteristics of pregnant women (*n* = 1717).

Characteristics	Mean ± SD or *n* (%)
Age (years)	27.01 ± 5.01
Pre-pregnancy BMI (kg/m^2^)	22.24 ± 3.64
Gestational weeks	36.44 ± 4.15
Education	
Junior high school or below	1028 (59.9)
Senior high school	453 (26.4)
College or above	236 (13.7)
Occupation	
Employed	1061 (61.8)
Unemployed	656 (38.2)
Family annual income	
<¥100,000 ($ 14,200)	429 (25.0)
100,000–150,000 CNY ($ 14,200–$ 21,300)	801 (46.7)
>150,000 CNY ($ 21,300)	404 (23.5)
Unknown	83 (4.8)
Passive smoking during pregnancy	
No	1480 (86.2)
Yes	237 (13.8)
Drinking during pregnancy	
No	1682 (98.0)
Yes	35 (2.0)
Physical activity before pregnancy	
No	1231 (71.7)
Yes	486 (28.3)
Nutrition supplementation during pregnancy	
No	103 (6.0)
Yes	1614 (94.0)
Dietary iron intake (g/week)	0.10 ± 0.02
Dietary calcium intake (g/week)	3.73 ± 0.87
Iron deficiency	
No	1334 (77.7)
Yes	383 (22.3)

**Table 2:** Basic characteristics of pregnant women who did not meet the inclusion criteria (*n* = 2529).

Characteristics	Mean ± SD or *n* (%)
Age (years)	27.23 ± 4.94
Pre-pregnancy BMI (kg/m^2^)	22.05 ± 3.55
Gestational weeks	36.54 ± 4.21
Education	
Junior high school or below	1273 (50.3)
Senior high school	656 (25.9)
College or above	600 (23.7)
Occupation	
Employed	1445 (57.1)
Unemployed	1084 (42.9)
Family annual income	
<¥100,000 ($ 14,200)	1196 (47.3)
100,000–150,000 CNY ($ 14,200–$ 21,300)	667 (26.4)
>150,000 CNY ($ 21,300)	372 (14.7)
Unknown	294 (11.6)
Passive smoking during pregnancy	
No	1839 (72.7)
Yes	690 (27.3)
Drinking during pregnancy	
No	2464 (97.4)
Yes	65 (2.6)
Physical activity before pregnancy	
No	1539 (60.9)
Yes	990 (39.1)
Nutrition supplementation during pregnancy	
No	114 (4.5)
Yes	2415 (95.5)
Dietary iron intake (g/week) ^①^	0.10 ± 0.02
Dietary calcium intake (g/week) ^①^	3.65 ± 0.91
Iron deficiency	
No	2185 (86.4)
Yes	344 (13.6)

① Estimated from the food frequency questionnaire; see Section 2.5 for the calculation.

## Data Availability

The datasets (de-identified questionnaire data, urinary PAH metabolite measurements, serum ferritin results, and R analysis code) are available from the corresponding author upon reasonable request. The data are not publicly available due to the privacy of pregnant women’s health information and data-governance rules of the participating hospitals and the Ethics Committee of Zunyi Medical University. Requests with a description of intended use can be submitted to Yuanzhong Zhou: zhouyuanzhong@zmu.edu.cn for review by the corresponding author and the Ethics Committee.

## Supplementary Materials

The following supporting information can be downloaded at https://www.mdpi.com/article/10.3390/nu18213565/s1. Figure S1. Forest plot of the association between urinary PAH metabolites and gestational iron deficiency (T3 vs T1) under four urine dilution-correction approaches: creatinine-normalized (primary), raw concentrations, raw concentrations with creatinine as a covariate, and specific-gravity-corrected concentrations. OR, odds ratio; CI, confidence interval.; Figure S2. Spearman correlations among urinary concentrations of the ten PAH metabolites; Table S1a. Adjusted odds ratios (95% CIs) of iron deficiency across tertiles of PAH metabolites using three imputation methods (LOD/√2, weighted regression, and random imputation); Table S1b. Logistic regression of binary PAH detection status on gestational iron deficiency; Table S1c. Sensitivity analyses: ORs with hospital site or grilled/smoked food frequency adjustment; Table S2. Nonlinearity testing for dose–response relationships between PAH metabolites and iron deficiency using restricted cubic spline models; Figure S3. Single-exposure dose–response function plot; Figure S4. Pairwise joint marginal effect plot; Figure S5. BKMR bivariate analysis: interaction pattern between 1-OHPyr and 3-OHPhe; Table S3a. Distributions of urinary PAH metabolite concentrations for pregnant women; Table S3b. Retention time, ion pair, collision energy, determination coefficient, recovery rate, and detection limit of each metabolite; Table S4. Distribution of stabilized inverse probability weights by LOD handling method; Table S5. Covariate balance and effective sample size before and after IPW; Table S6. E-values for single-pollutant and mixture associations.

## Abbreviations

PAHs	Polycyclic Aromatic Hydrocarbons
1-OHNap	1-hydroxynaphthalene
2-OHNap	2-hydroxynaphthalene
2-OHFlu	2-hydroxyfluorene
9-OHFlu	9-hydroxyfluorene
1-OHPhe	1-hydroxyphenanthrene
2-OHPhe	2-hydroxyphenanthrene
3-OHPhe	3-hydroxyphenanthrene
4-OHPhe	4-hydroxyphenanthrene
9-OHPhe	9-hydroxyphenanthrene
1-OHPyr	1-hydroxypyrene
IPW	Inverse Probability Weighting
RCS	Restricted Cubic Splines
BKMR	Bayesian Kernel Machine Regression
WQS	Weighted Quantile Sum
qgcomp/Q-g	quantile g-computation
OR	Odds Ratio
CI	Confidence Interval
GC–MS/MS	Gas chromatography–tandem mass spectrometry
LOD	Limit of Detection
S/N	signal-to-noise ratio
SG	Specific Gravity
FFQ	Food Frequency Questionnaire
IQR	Interquartile Range
MCMC	Markov Chain Monte Carlo
FDR	False Discovery Rate
PIP	Posterior Inclusion Probability
JAK2	Janus Kinase 2
STAT3	Signal Transducer and Activator of Transcription 3
CRP	C-Reactive Protein
OH-PAH	Hydroxylated Polycyclic Aromatic Hydrocarbon
