# Supplementary material for: Association of Polycyclic Aromatic Hydrocarbon Mixtures with Gestational Iron Deficiency: A Cross-Sectional Analysis of the Zunyi Birth Cohort
Source: Nutrients. 2026 Oct 28;18(21):3565. doi: 10.3390/nu18213565 (PMC13764953; doi:10.3390/nu18213565)
Supplement: Supplementary file 1 [file nutrients-18-03565-s001.zip › nutrients-4516398-SI.pdf]

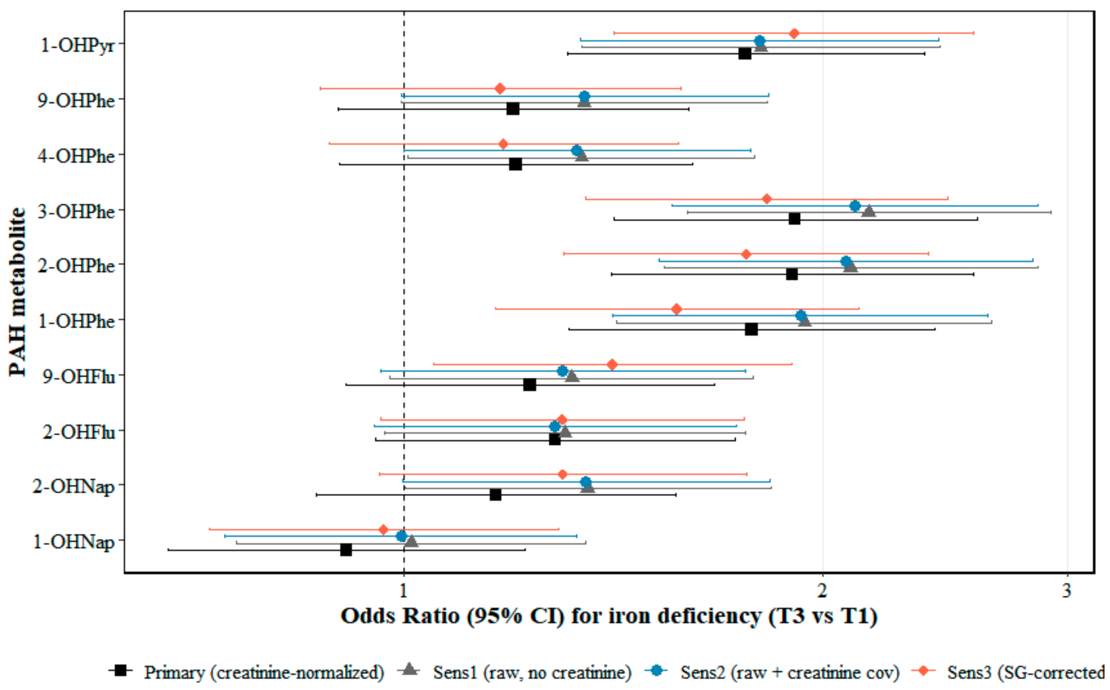

Figure S1. Forest plot of the association between urinary PAH metabolites and gestational iron deficiency (T3 vs T1) under four urine dilution-correction approaches: creatinine-normalized (primary), raw concentrations, raw concentrations with creatinine as a covariate, and specific-gravity-corrected concentrations. OR, odds ratio; CI, confidence interval.

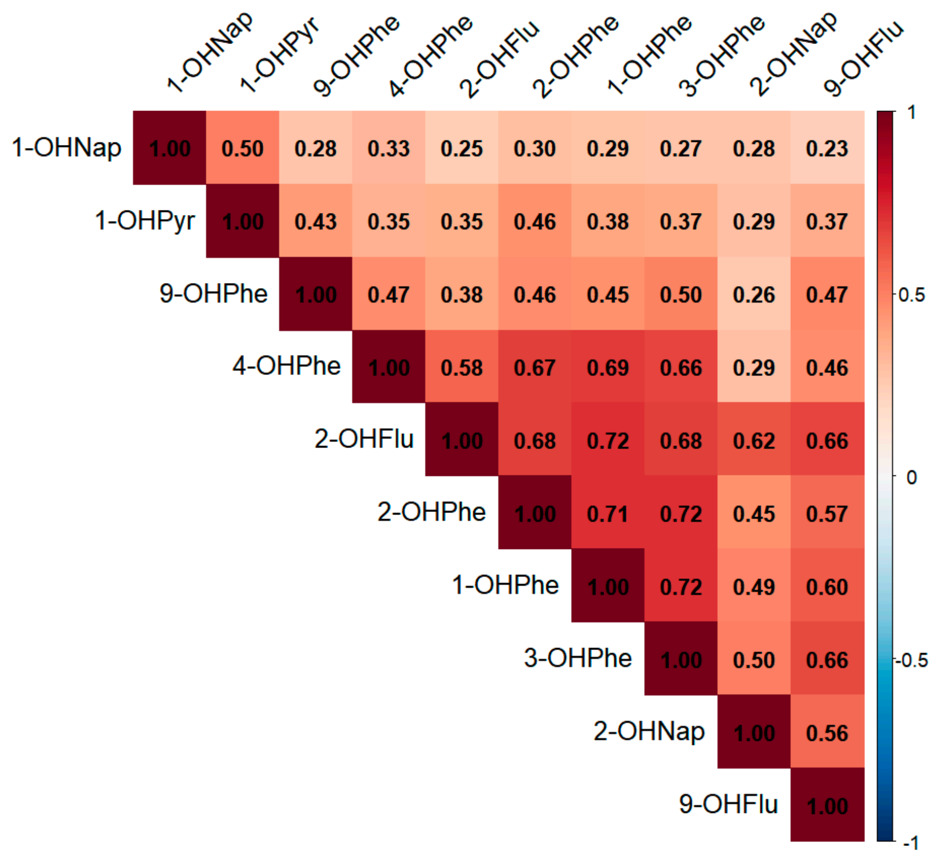

Figure S2. Spearman’s correlations among the urinary concentrations of 10 chemical exposures.

Table S1a. Adjusted odds ratios (95% CIs) of iron deficiency across tertiles of PAH metabolites using three imputation methods: LOD/√2, weighted regression, and random imputation

Method	Exposure	OR	CI_lower	CI_upper	P_trend	P_trend_FDR
LOD/√2 Imputation	1-OHNap	1.09064	0.82504	1.44174	0.52524	0.52524
LOD/√2 Imputation	1-OHNap	0.91138	0.68313	1.21590	0.52524	0.52524
LOD/√2 Imputation	2-OHNap	1.08301	0.80638	1.45455	0.20329	0.22588
LOD/√2 Imputation	2-OHNap	1.20908	0.90269	1.61945	0.20329	0.22588
LOD/√2 Imputation	2-OHFlu	1.34967	1.00465	1.81316	0.01780	0.03560
LOD/√2 Imputation	2-OHFlu	1.43038	1.06304	1.92467	0.01780	0.03560
LOD/√2 Imputation	9-OHFlu	1.57895	1.18351	2.10650	0.12713	0.18515
LOD/√2 Imputation	9-OHFlu	1.25678	0.93250	1.69382	0.12713	0.18515
LOD/√2 Imputation	1-OHPhe	1.48584	1.10123	2.00479	0.00006	0.00020
LOD/√2 Imputation	1-OHPhe	1.82655	1.35899	2.45499	0.00006	0.00020
LOD/√2 Imputation	2-OHPhe	1.41035	1.04343	1.90630	0.00001	0.00006
LOD/√2 Imputation	2-OHPhe	1.95779	1.46016	2.62502	0.00001	0.00006
LOD/√2 Imputation	3-OHPhe	1.40714	1.04190	1.90043	0.00001	0.00006
LOD/√2 Imputation	3-OHPhe	1.92312	1.43446	2.57825	0.00001	0.00006
LOD/√2 Imputation	4-OHPhe	1.10809	0.83069	1.47812	0.12961	0.18515
LOD/√2 Imputation	4-OHPhe	1.24711	0.93726	1.65938	0.12961	0.18515
LOD/√2 Imputation	9-OHPhe	0.92420	0.69346	1.23172	0.17229	0.21536
LOD/√2 Imputation	9-OHPhe	1.21912	0.91961	1.61618	0.17229	0.21536
LOD/√2 Imputation	1-OHPyr	1.21081	0.89926	1.63028	0.00096	0.00241
LOD/√2 Imputation	1-OHPyr	1.62528	1.21855	2.16776	0.00096	0.00241
Weighted Regression	1-OHNap	1.04347	0.78913	1.37977	0.44648	0.44648
Weighted Regression	1-OHNap	0.89487	0.67147	1.19259	0.44648	0.44648
Weighted Regression	2-OHNap	1.18021	0.87877	1.58506	0.10544	0.14086
Weighted Regression	2-OHNap	1.27208	0.94970	1.70390	0.10544	0.14086
Weighted Regression	2-OHFlu	1.37675	1.02482	1.84954	0.01081	0.02163
Weighted Regression	2-OHFlu	1.46950	1.09203	1.97744	0.01081	0.02163
Weighted Regression	9-OHFlu	1.55685	1.16654	2.07774	0.11269	0.14086
Weighted Regression	9-OHFlu	1.26832	0.94135	1.70886	0.11269	0.14086
Weighted Regression	1-OHPhe	1.38716	1.02827	1.87130	0.00019	0.00062
Weighted Regression	1-OHPhe	1.74961	1.30329	2.34877	0.00019	0.00062
Weighted Regression	2-OHPhe	1.33989	0.99143	1.81084	0.00001	0.00006
Weighted Regression	2-OHPhe	1.91535	1.43158	2.56259	0.00001	0.00006
Weighted Regression	3-OHPhe	1.37110	1.01645	1.84949	0.00001	0.00006
Weighted Regression	3-OHPhe	1.91445	1.43179	2.55981	0.00001	0.00006
Weighted Regression	4-OHPhe	1.20851	0.90510	1.61363	0.05791	0.09651
Weighted Regression	4-OHPhe	1.32142	0.99036	1.76315	0.05791	0.09651
Weighted Regression	9-OHPhe	0.92414	0.69378	1.23099	0.21974	0.24415
Weighted Regression	9-OHPhe	1.19516	0.90148	1.58451	0.21974	0.24415
Weighted Regression	1-OHPyr	1.21962	0.90610	1.64161	0.00137	0.00344
Weighted Regression	1-OHPyr	1.60267	1.20095	2.13878	0.00137	0.00344

Method	Exposure	OR	CI_lower	CI_upper	P_trend	P_trend_FDR
Random Imputation	1-OHNap	1.10757	0.83740	1.46492	0.64063	0.64063
Random Imputation	1-OHNap	0.93428	0.70022	1.24658	0.64063	0.64063
Random Imputation	2-OHNap	1.16921	0.87049	1.57045	0.11605	0.16578
Random Imputation	2-OHNap	1.26363	0.94300	1.69328	0.11605	0.16578
Random Imputation	2-OHFlu	1.32327	0.98540	1.77700	0.02176	0.04352
Random Imputation	2-OHFlu	1.41345	1.05101	1.90087	0.02176	0.04352
Random Imputation	9-OHFlu	1.55261	1.16324	2.07232	0.08922	0.14870
Random Imputation	9-OHFlu	1.28995	0.95795	1.73702	0.08922	0.14870
Random Imputation	1-OHPhe	1.43497	1.06429	1.93475	0.00013	0.00043
Random Imputation	1-OHPhe	1.77405	1.32137	2.38181	0.00013	0.00043
Random Imputation	2-OHPhe	1.41289	1.04643	1.90770	0.00001	0.00011
Random Imputation	2-OHPhe	1.92982	1.43919	2.58770	0.00001	0.00011
Random Imputation	3-OHPhe	1.41132	1.04495	1.90612	0.00002	0.00012
Random Imputation	3-OHPhe	1.88177	1.40275	2.52437	0.00002	0.00012
Random Imputation	4-OHPhe	1.08701	0.81464	1.45043	0.16188	0.20234
Random Imputation	4-OHPhe	1.22598	0.92150	1.63106	0.16188	0.20234
Random Imputation	9-OHPhe	0.92161	0.69148	1.22832	0.18617	0.20685
Random Imputation	9-OHPhe	1.21127	0.91403	1.60517	0.18617	0.20685
Random Imputation	1-OHPyr	1.26250	0.93736	1.70043	0.00067	0.00168
Random Imputation	1-OHPyr	1.65311	1.23778	2.20781	0.00067	0.00168

Note: *All models were adjusted for the same set of covariates: [age, education, occupation, family annual income, physical activity before pregnancy, drinking during pregnancy, passive smoking during pregnancy, nutritional supplementation, BMI, iron intake and gestational weeks during pregnancy].*

Table S1b. Logistic regression of binary PAH detection status on gestational iron deficiency .

Metabolite	Detected n	Non-detected n	OR (95% CI)	P	FDR
1-OHPyr	1132	585	1.50 (1.15–1.94)	0.002	0.006
9-OHPhe	1040	677	1.31 (1.02–1.68)	0.036	0.052
4-OHPhe	845	872	1.27 (0.99–1.61)	0.057	0.071
3-OHPhe	1047	670	1.52 (1.18–1.96)	0.001	0.004
2-OHPhe	966	751	1.75 (1.36–2.25)	<0.001	<0.001
1-OHPhe	1012	705	1.69 (1.31–2.19)	<0.001	<0.001
9-OHFlu	1216	501	1.53 (1.16–2.01)	0.003	0.006
2-OHFlu	1315	402	1.57 (1.16–2.12)	0.003	0.006
2-OHNap	878	839	1.25 (0.97–1.59)	0.082	0.091
1-OHNap	1068	649	0.97 (0.76–1.23)	0.782	0.782

Table S1c. Sensitivity analyses: ORs with hospital or barbecue frequency adjustment.

Metabolite	Primary analysis	+ Hospital	+ Barbecue frequency
1-OHNap	0.91 (0.68–1.22)	0.92 (0.69–1.23)	0.90 (0.68–1.21)
2-OHNap	1.21 (0.90–1.62)	1.25 (0.93–1.67)	1.23 (0.92–1.65)
2-OHFlu	1.43 (1.06–1.92)	1.48 (1.10–2.00)	1.46 (1.08–1.96)
9-OHFlu	1.26 (0.93–1.69)	1.36 (1.01–1.84)	1.26 (0.93–1.69)
1-OHPhe	1.83 (1.36–2.45)	1.91 (1.42–2.56)	1.86 (1.39–2.51)
2-OHPhe	1.96 (1.46–2.63)	2.02 (1.51–2.71)	1.98 (1.48–2.66)
3-OHPhe	1.92 (1.43–2.58)	1.95 (1.45–2.61)	1.94 (1.45–2.61)
4-OHPhe	1.25 (0.94–1.66)	1.27 (0.95–1.69)	1.28 (0.96–1.71)
9-OHPhe	1.22 (0.92–1.62)	1.37 (1.03–1.82)	1.23 (0.92–1.63)
1-OHPyr	1.63 (1.22–2.17)	1.72 (1.29–2.30)	1.61 (1.21–2.15)

Note: *Odds ratios compare the highest tertile (T3) versus the lowest tertile (T1) of each PAH metabolite. Primary model adjusted for maternal age, pre-pregnancy BMI, education, occupation, income, passive smoking, exercise, drinking, nutritional supplementation, iron intake, and gestational week. Hospital-adjusted model additionally included hospital site. Barbecue-adjusted model additionally included grilled/smoked food intake frequency. All models used stabilized IPW and LOD/√2 imputation.*

Table S2. Nonlinearity testing for dose-response relationships between PAH metabolites and iron deficiency using restricted cubic spline models.

Metabolite	Overall χ^2 (2 df)	Overall P-value	Nonlinear χ^2 (1 df)	Nonlinear P-value	Conclusion
1-OHNap	1.80	0.4070	1.11	0.2912	No significant association
2-OHNap	2.18	0.3355	0.35	0.5522	No significant association
**2-OHFlu**	**14.61**	**0.0007**	**12.01**	**0.0005**	Significant nonlinear association
**9-OHFlu**	**14.23**	**0.0008**	**11.57**	**0.0007**	Significant nonlinear association
**1-OHPhe**	**9.96**	**0.0069**	1.13	0.2875	Significant linear association
**2-OHPhe**	**12.46**	**0.0020**	3.41	0.0649	Significant linear association
**3-OHPhe**	**11.95**	**0.0025**	3.84	0.0501	Significant linear association (borderline nonlinear)
4-OHPhe	1.75	0.4171	1.60	0.2061	No significant association
9-OHPhe	0.29	0.8644	0.25	0.6176	No significant association
**1-OHPyr**	**11.25**	**0.0036**	0.07	0.7982	Significant linear association

Abbreviations: *df, degrees of freedom.*

Note: *Statistically significant results (P < 0.05) are shown in bold. All models were adjusted for maternal age (RCS),*

pre-pregnancy BMI (RCS), dietary iron intake (RCS), education, occupation, household income, passive smoking, physical activity, alcohol consumption, and nutritional status.

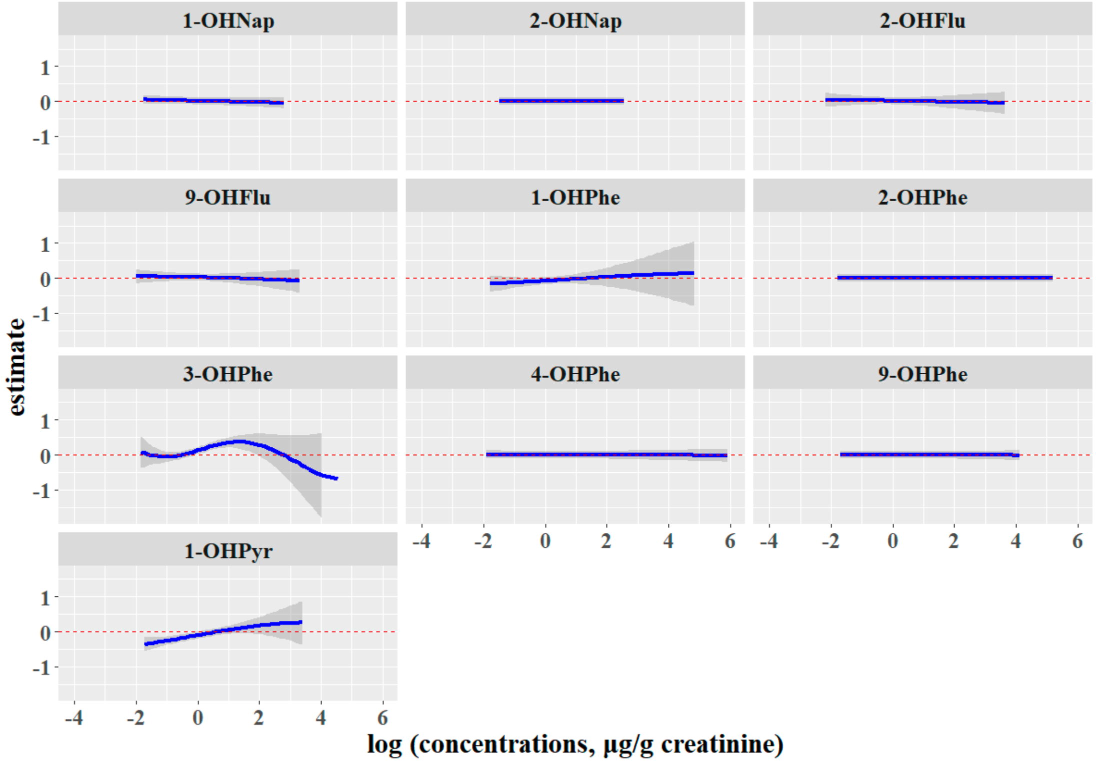

Figure S3. Single-exposure dose-response function plot

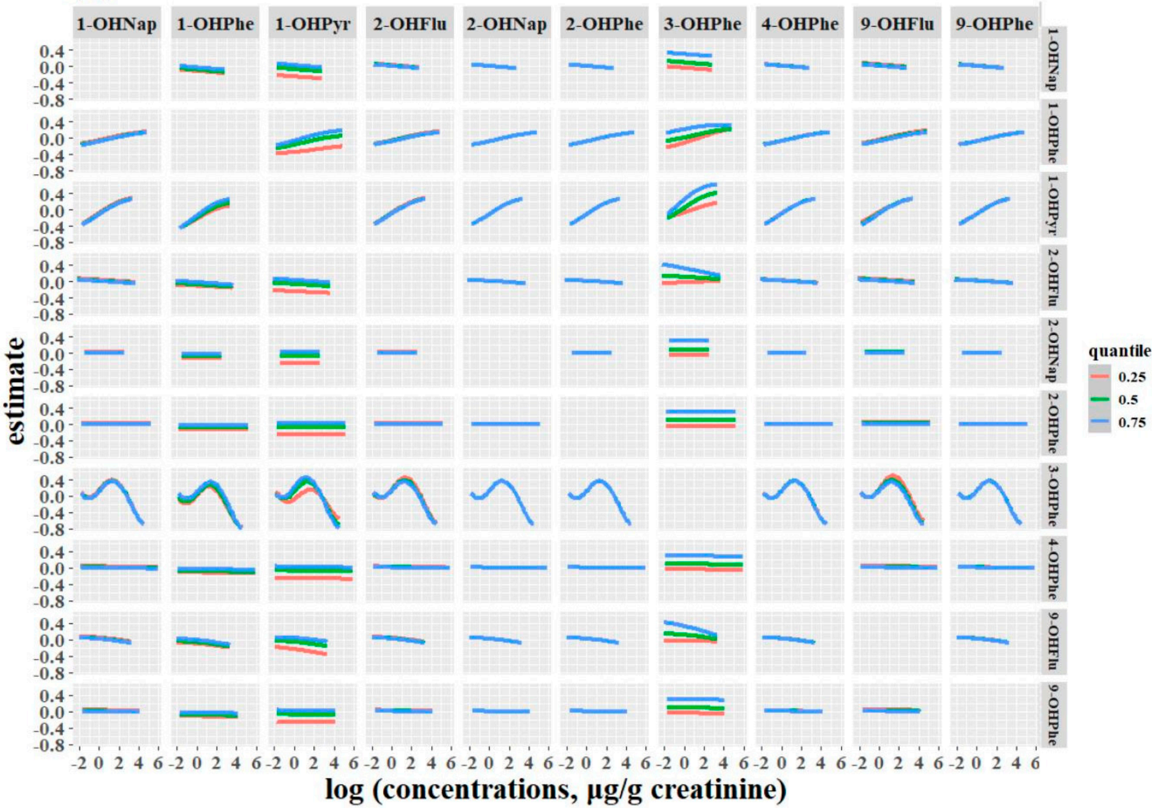

Figure S4. Pairwise joint marginal effect plot

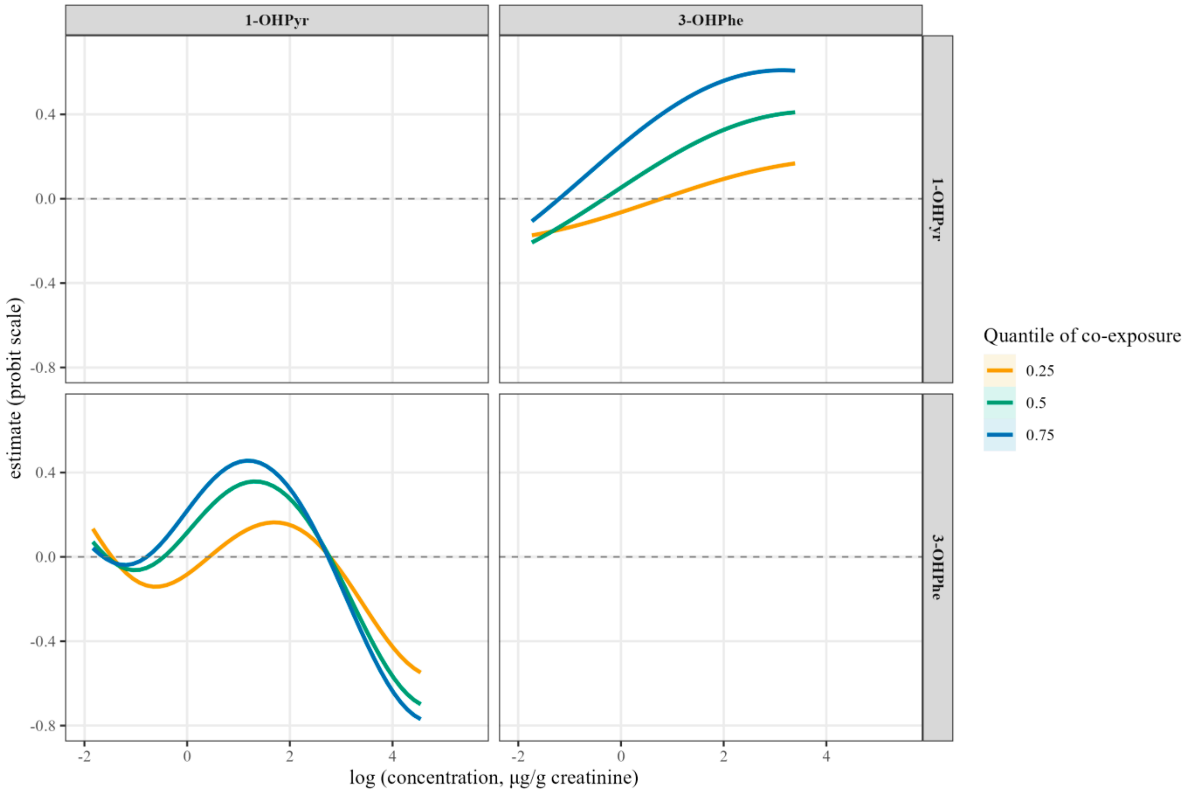

Figure S5. BKMR bivariate analysis: interaction pattern between 1-OHPyr and 3-OHPhe

Analyte	LOD	DR (%)	Creatinine-adjusted (µg/g creatinine)	Unadjusted (µg/L)

Table S3a. Distributions of urinary PAHs metabolites concentrations for pregnant women.

			Medi an	25th percentile	75th percentile	Range	Median	25th percentile	75th percentile	Range
1-OHNap	0.0050	62.2	0.343	0.008	1.473	0.0009-479.5327	0.290	0.004	1.046	0.0035-153.2412
2-OHNap	0.0027	51.1	0.042	0.003	1.382	0.0005-347.6331	0.033	0.002	1.038	0.0019-111.0909
2-OHFlu	0.0167	76.6	0.217	0.019	0.719	0.0005-1094.8904	0.156	0.014	0.508	0.0021-936.4655
9-OHFlu	0.0044	70.8	0.302	0.006	1.077	0.0004-1430.2110	0.221	0.002	0.742	0.0016-1223.267
1-OHPhe	0.0029	58.9	0.035	0.008	0.203	0.0012-702.1864	0.025	0.005	0.136	0.0050-11.9037
2-OHPhe	0.0023	56.3	0.039	0.012	0.180	0.0020-751.7273	0.021	0.008	0.125	0.0081-642.9563
3-OHPhe	0.0071	61.0	0.039	0.011	0.184	0.0017-243.9267	0.027	0.007	0.132	0.0067-193.6383
4-OHPhe	0.0115	49.2	0.035	0.015	0.098	0.0029-175.1325	0.012	0.012	0.064	0.0118-66.9294
9-OHPhe	0.0095	60.6	0.017	0.005	0.093	0.0008-121.5769	0.010	0.003	0.064	0.0031-35.9919
1-OHPyr	0.0003	65.9	0.017	0.001	0.101	0.0001-322.6590	0.011	0.000	0.072	0.0002-14.7913

LOD: *limit of detection; DR: detection rates.*

Table S3b. Retention time, ion pair, collision energy, determination coefficient, recovery rate, and detection limit of each metabolite.

**Chemical**	**RT (min)**	**Precursor Ion**	**Product Ion**	**CE (eV)**	**ISTD**	**Determination coefficient (R^2)**	**LOD (µg /L)**	**Recovery (%)**	**RSD (%)**
1-OHNap-D7	15.987	223	208	15	N/A	N/A	N/A	N/A	N/A
1-OHNap	16.043	216	201	15	1-OHNap-D7	0.9998	0.0050	107.05	1.4
2-OHNap	16.547	216	201	15	1-OHNap-D7	0.9999	0.0027	105.71	2.27
9-OHFlu	20.231	254	165	20	1-OHNap-D7	0.9998	0.0023	94.15	2.85
2-OHFlu	22.471	254	239	15	1-OHNap-D7	0.9998	0.0029	91.99	3.18
4-OHPhe	25.209	266	235	25	1-OHNap-D7	0.9999	0.0167	90.08	5.01
9-OHPhe	25.211	266	73	25	1-OHNap-D7	0.9982	0.0044	105.01	4.99
1-OHPhe	25.685	266	73	25	1-OHNap-D7	0.9998	0.0071	81.09	4.96
3-OHPhe	25.854	266	73	25	1-OHNap-D7	0.9999	0.0095	84.5	5.48
2-OHPhe	26.702	266	73	25	1-OHNap-D7	0.9999	0.0115	68.19	2.41
1-OHPyr	30.857	290	73	25	1-OHPyr-D9	0.9999	0.0003	100.3	1.97
1-OHPyr-D9	31.229	299	73	25	N/A	N/A	N/A	N/A	N/A

RT: *retention time; CE: collision energy; ISTD: internal standard substance; LOD: limit of detection; RSD: relative standard deviation; N/A: not available.*

Table S4. Distribution of stabilized inverse probability weights and effective sample size, by metabolite and LOD handling method

Metabolite	Method	Min	P1	Q1	Median	Q3	P99	Max	ESS	ESS/N (%)
1-OHNap	LOD/√2 Imputation	0.586	0.586	0.879	0.974	1.092	1.667	1.667	1657.1	96.5
2-OHNap	LOD/√2 Imputation	0.523	0.524	0.806	0.945	1.122	2.010	2.012	1594.6	92.9
2-OHFlu	LOD/√2 Imputation	0.503	0.504	0.834	0.948	1.091	2.225	2.225	1597.9	93.1
9-OHFlu	LOD/√2 Imputation	0.426	0.426	0.875	0.969	1.074	1.915	1.917	1639.6	95.5
1-OHPhe	LOD/√2 Imputation	0.536	0.536	0.850	0.963	1.097	1.783	1.791	1631.3	95.0
2-OHPhe	LOD/√2 Imputation	0.549	0.550	0.836	0.963	1.097	2.091	2.096	1614.4	94.0
3-OHPhe	LOD/√2 Imputation	0.585	0.585	0.860	0.973	1.086	1.786	1.787	1649.0	96.0
4-OHPhe	LOD/√2 Imputation	0.650	0.650	0.844	0.979	1.118	1.655	1.656	1650.5	96.1
9-OHPhe	LOD/√2 Imputation	0.546	0.547	0.867	0.974	1.089	1.810	1.816	1642.7	95.7
1-OHPyr	LOD/√2 Imputation	0.495	0.495	0.809	0.966	1.143	1.986	1.986	1598.7	93.1
1-OHNap	Weighted Regression	0.590	0.592	0.869	0.974	1.100	1.704	1.711	1655.3	96.4
2-OHNap	Weighted Regression	0.514	0.515	0.803	0.946	1.118	1.991	1.994	1595.3	92.9
2-OHFlu	Weighted Regression	0.510	0.511	0.830	0.951	1.089	2.227	2.236	1601.1	93.3
9-OHFlu	Weighted Regression	0.426	0.426	0.875	0.968	1.066	1.916	1.917	1639.3	95.5
1-OHPhe	Weighted Regression	0.533	0.533	0.848	0.965	1.100	1.776	1.779	1626.9	94.8
2-OHPhe	Weighted Regression	0.558	0.558	0.833	0.958	1.099	1.995	2.005	1618.8	94.3
3-OHPhe	Weighted Regression	0.608	0.608	0.862	0.966	1.084	1.700	1.701	1652.8	96.3
4-OHPhe	Weighted Regression	0.651	0.651	0.836	0.982	1.120	1.621	1.621	1649.9	96.1
9-OHPhe	Weighted Regression	0.548	0.548	0.864	0.976	1.087	1.818	1.822	1642.5	95.7
1-OHPyr	Weighted Regression	0.497	0.497	0.812	0.964	1.137	1.999	2.003	1602.8	93.4
1-OHNap	Random Imputation	0.581	0.583	0.877	0.971	1.097	1.670	1.670	1655.4	96.4
2-OHNap	Random Imputation	0.538	0.538	0.802	0.941	1.132	1.988	1.993	1597.9	93.1
2-OHFlu	Random Imputation	0.504	0.505	0.836	0.951	1.090	2.230	2.235	1598.7	93.1
9-OHFlu	Random Imputation	0.426	0.426	0.876	0.965	1.074	1.913	1.913	1641.1	95.6
1-OHPhe	Random Imputation	0.536	0.536	0.856	0.962	1.091	1.843	1.843	1629.9	94.9
2-OHPhe	Random Imputation	0.559	0.559	0.845	0.963	1.089	2.108	2.111	1616.6	94.2
3-OHPhe	Random Imputation	0.574	0.574	0.858	0.967	1.084	1.800	1.801	1646.2	95.9
4-OHPhe	Random Imputation	0.654	0.654	0.839	0.979	1.123	1.700	1.701	1645.5	95.8
9-OHPhe	Random Imputation	0.554	0.555	0.861	0.974	1.088	1.810	1.812	1641.6	95.6
1-OHPyr	Random Imputation	0.505	0.505	0.807	0.963	1.142	1.994	2.000	1597.7	93.0

Note: *N/A: not available.Note: P1/P99 = 1st/99th percentiles; Q1/Q3 = 25th/75th percentiles. ESS = effective sample size =* $(\Sigma w)^2/\Sigma w^2$*; ESS/N = ESS as a percentage of the original sample n=*1,717*. Weights were winsorized at the 1st and 99th percentiles (about 36 participants, ~2%, truncated in each fit). A separate weight was estimated for each metabolite and each LOD handling method (30 fits in total); before truncation the largest weight reached 14.3.*

Table S5. Maximum covariate standardized mean difference before and after IPW, by metabolite and LOD handling method

Metabolite	Method	Max SMD (before weighting)	Max SMD (after weighting)
1-OHNap	LOD/√2 Imputation	0.231	0.056
2-OHNap	LOD/√2 Imputation	0.398	0.218
2-OHFlu	LOD/√2 Imputation	0.333	0.066
9-OHFlu	LOD/√2 Imputation	0.417	0.130
1-OHPhe	LOD/√2 Imputation	0.386	0.211
2-OHPhe	LOD/√2 Imputation	0.346	0.142
3-OHPhe	LOD/√2 Imputation	0.352	0.186
4-OHPhe	LOD/√2 Imputation	0.282	0.020
9-OHPhe	LOD/√2 Imputation	0.351	0.161
1-OHPyr	LOD/√2 Imputation	0.318	0.041
1-OHNap	Weighted Regression	0.252	0.054
2-OHNap	Weighted Regression	0.403	0.219
2-OHFlu	Weighted Regression	0.344	0.090
9-OHFlu	Weighted Regression	0.417	0.130
1-OHPhe	Weighted Regression	0.386	0.213
2-OHPhe	Weighted Regression	0.335	0.148
3-OHPhe	Weighted Regression	0.354	0.215
4-OHPhe	Weighted Regression	0.282	0.026
9-OHPhe	Weighted Regression	0.351	0.159
1-OHPyr	Weighted Regression	0.312	0.038
1-OHNap	Random Imputation	0.244	0.057
2-OHNap	Random Imputation	0.393	0.219
2-OHFlu	Random Imputation	0.333	0.065

Metabolite	Method	Max SMD (before weighting)	Max SMD (after weighting)
9-OHFlu	Random Imputation	0.417	0.131
1-OHPhe	Random Imputation	0.374	0.184
2-OHPhe	Random Imputation	0.328	0.119
3-OHPhe	Random Imputation	0.358	0.186
4-OHPhe	Random Imputation	0.285	0.023
9-OHPhe	Random Imputation	0.346	0.160
1-OHPyr	Random Imputation	0.316	0.040

Note: *SMD = standardized mean difference. For each fit the table reports the maximum absolute SMD across the 11 covariates (age, BMI, education, employment, income, passive smoking, exercise, drinking, nutrition, iron intake, gestational age) before and after weighting. In the primary LOD/√2 analysis the overall maximum across the ten metabolites was 0.417 (9-OHFlu) before and 0.218 (2-OHNap) after weighting—the 0.42 and 0.22 quoted in the main text; these two extrema come from different metabolites. Per-covariate SMDs for all 30 fits are provided in the accompanying IPW_Balance_SMD_All_Methods.csv.*

Table S6. E-values for single-pollutant and mixture associations

Analysis	OR (95% CI)	E-value, point	E-value, CI bound
1-OHPhe	1.83 (1.36–2.45)	2.04	1.61
2-OHPhe	1.96 (1.46–2.63)	2.15	1.71
3-OHPhe	1.92 (1.43–2.58)	2.12	1.68
1-OHPyr	1.63 (1.22–2.17)	1.87	1.44
2-OHFlu	1.43 (1.06–1.92)	1.68	1.20
Quantile g-computation	1.14 (1.01–1.29)	1.34	1.06
WQS	1.32 (1.11–1.57)	1.56	1.29

Note: *E-values indicate the minimum strength of joint association (on the risk-ratio scale) that an unmeasured confounder would need to have with both PAH exposure and iron deficiency to move an estimate to the null; larger values indicate greater robustness. Because the overall iron-deficiency prevalence (22.3%) exceeded 15%, odds ratios were first converted to the risk-ratio scale using* $RR \approx \sqrt{OR}$*, and E-values were then computed as* $E = RR + \sqrt{RR(RR - 1)}$*. The "CI bound closest to null" uses the lower confidence limit for these harmful estimates. Single-pollutant estimates are odds ratios for T3 vs T1; the mixture estimate is exp(ψ) for quantile g-computation and corresponds to a one-quartile increase in the mixture.*
